# Cattaraugus County Medical Society

**Published:** 1888-01

**Authors:** Lyman Dick

**Affiliations:** Secretary


### *CATTARAUGUS COUNTY MEDICAL SOCIETY.*

The semi-annual meeting of the Cattaraugus County Medical Society was held at the rooms of Dr. Lyman Dick, Salamanca, N. Y., December 7, 1887. The meeting was called to order by the President, Dr. O. A. Tompkins, of Randolph, N. Y.

The minutes of the last meeting were read and approved. The Secretary was authorized to publish a list of the names of all the physicians registered at the County Clerk's office, and to mail a copy to each member of the Society. The names of eight physicians were presented for admission, and by unanimous vote all were elected members of the Society. Dr. V. A. Ellsworth, of East Otto, N. Y., was elected delegate to the State Medical Society. Drs. A. A. Hubbell and H. D. Ingraham, of the Medical Department of Niagara University, Buffalo, N. Y., were present by special invitation.

Dr. Ingraham read a very interesting paper on "Some Points in Abdominal Surgery." He spoke more particularly on abdominal section, and urged the proper preparation of the patient, of the operating-room, and of the instruments to be used, before the operation, and proper diet, nursing and after-treatment after the operation.

Dr. Ingraham also presented, by request, O'Dwyer's instruments for intubation of the larynx, and described the methods of using them. The Doctor had had quite a large experience in their use in membranous croup, and while he had not been very successful with them in saving life, yet he advised their use, as the patients were thus enabled to breathe easily, and were made comfortable. In some cases, they no doubt saved life, although in this respect he had, so far, failed.

Dr. Hubbell read an interesting paper on "Eye Symptoms in Disease." He referred to those eye troubles which were functional, and those which showed local structural lesions that might be looked upon as important symptoms of diseases elsewhere in the body. Paralytic and spasmodic affections of the extra-ocular and intra-ocular muscles were dwelt upon, and their significance pointed out. The indications of the state of vision were also referred to as suggestive of diseases of the nervous system. He concluded with a brief description of those eye diseases exhibiting structural changes locally, and their symptomatic relation to other diseases. He illustrated his subject with several beautiful plates, and with diagrams.

DR. DICK gave a microscopical exhibition of the "Bacillus Tuberculosis," and demonstrated the rapid method of staining, according to the recent formula of Prof. Burrill.

Other papers were presented, but they were not read, owing to the limited time.

The reading of the papers was followed by discussion, after which the meeting adjourned to meet again on the first Wednesday of June, 1888.

The attendance was unusually large, and the meeting was pleasant and satisfactory.

LYMAN DICK,
*Secretary.*

